# Prognostic value of circulating tumour cells in limited-stage small-cell lung cancer: analysis of the concurrent once-daily versus twice-daily radiotherapy (CONVERT) randomised controlled trial

**DOI:** 10.1093/annonc/mdz122

**Published:** 2019-04-24

**Authors:** R Y Tay, F Fernández-Gutiérrez, V Foy, K Burns, J Pierce, K Morris, L Priest, J Tugwood, L Ashcroft, C R Lindsay, C Faivre-Finn, C Dive, F Blackhall

**Affiliations:** 1 Department of Medical Oncology, The Christie NHS Foundation Trust, Manchester; 2 Clinical and Experimental Pharmacology Group, CRUK Manchester Institute; 3 Division of Molecular and Clinical Cancer Sciences, School of Medical Sciences, Faculty of Biology, Medicine and Health; 4 Cancer Research UK Manchester Institute; 5 Manchester Centre for Cancer Biomarker Sciences, University of Manchester, Manchester; 6 Manchester Academic Health Science Centre Trials Co-ordination Unit; 7 Department of Radiotherapy Related Research, The Christie NHS Foundation Trust, Manchester, UK

**Keywords:** small-cell lung cancer, limited disease, limited stage, circulating tumour cells, chemoradiotherapy, prognosis

## Abstract

**Background:**

The clinical significance of circulating tumour cells (CTCs) in limited-stage small-cell lung cancer (LS-SCLC) is not well defined. We report a planned exploratory analysis of the prevalence and prognostic value of CTCs in LS-SCLC patients enrolled within the phase III randomised CONVERT (concurrent once-daily versus twice-daily chemoradiotherapy) trial.

**Patients and methods:**

Baseline blood samples were enumerated for CTCs using CellSearch in 75 patients with LS-SCLC who were enrolled in the CONVERT trial and randomised between twice- and once-daily concurrent chemoradiation. Standard statistical methods were used for correlations of CTCs with clinical factors. Log-rank test and Cox regression analyses were applied to establish the associations of 2, 15 and 50 CTC thresholds with progression-free survival (PFS) and overall survival (OS). An optimal CTC count threshold for LS-SCLC was established.

**Results:**

CTCs were detected in 60% (45/75) of patients (range 0–3750). CTC count thresholds of 2, 15 and 50 CTCs all significantly correlate with PFS and OS. An optimal CTC count threshold in LS-SCLC was established at 15 CTCs, defining ‘favourable’ and ‘unfavourable’ prognostic risk groups. The median OS in <15 versus ≥15 CTCs was 26.7 versus 5.9 m (*P* = 0.001). The presence of ≥15 CTCs at baseline independently predicted ≤1 year survival in 70% and ≤2 years survival in 100% of patients.

**Conclusion:**

We report the prognostic value of baseline CTC count in an exclusive LS-SCLC population at thresholds of 2, 15 and 50 CTCs. Specific to LS-SCLC, ≥15 CTCs was associated with worse PFS and OS independent of all other factors and predicted ≤2 years survival. These results may improve disease stratification in future clinical trial designs and aid clinical decision making.

**Trial registration:**

ClinicalTrials.gov identifier: NCT00433563.

**Key Message**

We report on the prognostic value of baseline CTCs in LS-SCLC. CTC count thresholds of 2, 15 and 50 CTCs all significantly correlate with PFS and OS. Greater than 15 CTCs at baseline defines an ‘unfavourable’ prognostic risk group, predicting ≤1 year survival in 70% and ≤2 years survival in 100% of patients. CTC count has utility for treatment stratification and decision making in LS-SCLC.

## Introduction 

Lung cancer remains the leading cause of mortality worldwide [1]. Small**-**cell lung cancer (SCLC) accounts for 13% of lung cancer incidence and is characterised by rapid doubling-time, propensity for early metastasis, and high rates of relapse and resistant disease. One-third of patients will present with limited-stage SCLC (LS-SCLC) [2, 3], with concurrent chemoradiotherapy (CCRT) offering the best chance of cure in LS-SCLC patients with good performance status (PS) [4]. Despite this aggressive treatment, a high proportion of LS-SCLC patients subsequently relapse.

Circulating tumour cells (CTCs) represent a novel method of non-invasively evaluating real-time disease biology. CTC enumeration, molecular characterisation and dynamic CTC monitoring are under evaluation in a number of cancers including breast, castrate-resistant prostate cancer (CRPC), colorectal and non-SCLC (NSCLC) [5]. As a prognostic marker, CTC enumeration is validated in metastatic breast, CRPC, colorectal and NSCLC [6–9]. In SCLC, CTC count thresholds of 2 CTCs [10, 11] and 50 CTCs [12] have been previously demonstrated to be prognostic [12], however, these analyses were carried out in heterogeneous patient populations that included both extensive-stage (ES) and LS patients treated as standard of care.

The phase III CONVERT (concurrent once-daily versus twice-daily chemoradiotherapy in patients with LS-SCLC, NCT00433563) study randomised 547 patients with LS-SCLC to twice-daily CCRT or once-daily CCRT [13]. The study affirmed the twice-daily CCRT regimen as standard of care. Median progression**-**free survival (PFS) was 14.3–15.4 months with 2- and 5-year overall survival (OS) rates of 51%–56% and 31%–34%, respectively [13]. At present, there are no routinely used objective methods that identify the patients with LS-SCLC who are at greatest risk of early relapse nor those who will achieve long-term response and cure.

The CONVERT trial provided opportunity to further define the clinical significance of CTCs in an exclusively LS population, within a phase III, randomised controlled, clinical trial.

## Methods

### Study design and procedures

Full details of the CONVERT trial design were previously published [13, 14]. Eligible patients were ≥18 years with histologically or cytological-confirmed LS-SCLC and Eastern Cooperative Oncology Group (ECOG) PS 0–1. Patients with ECOG PS 2 due to cancer-related symptoms were included at the discretion of the local investigator. Staging FDG-PET was allowed, but not mandated. All CONVERT patients recruited in Manchester, UK had blood samples collected for CTC enumeration. Blood samples (7.5 ml) were collected before commencement of any treatment. CTC enumeration was carried out using the CellSearch platform as previously described [15, 16]. A CTC was defined by co-expression of transmembrane glycoprotein epithelial cell adhesion molecule and cytokeratin 8, 18 and 19 in the absence of CD45 expression. The REMARK guidelines for reporting prognostic biomarker study results have been followed [17].

### Statistical analysis

Associations of CTCs with clinical characteristics were studied using Fisher’s exact and Mann–Whitney tests. Correlation of CTC count with gross tumour volume (GTV) at baseline was compared using Spearman’s *ρ* analysis. CTC count distribution was compared within TNM stage groups (group I: TNM stage I and II and group II: TNM stage IIIA-B) and PS (0, 1 and 2), using the Mann–Whitney and Kruskal–Wallis tests, respectively.

The validity of previously published SCLC CTC count thresholds of 2 and 50 CTCs was assessed using Kaplan–Meier analysis and log-rank tests for PFS and OS. PFS was defined from date of randomisation to date of first clinical or radiological evidence of progressive disease at the primary site or distant sites. OS was defined as time from randomisation until death from any cause. As previous series enrolled LS- and ES-SCLC, an optimal prognostic CTC threshold in an exclusively LS-SCLC population was defined as the highest cut-off point correlating with OS using the Kaplan–Meier log-rank test with Bonferroni correction and highest area under the receiver operating characteristic curve for predicting 1-year OS.

Univariate Cox proportional hazard regression analysis for OS and PFS was carried out for 2, 15 and 50 CTC count thresholds, PET staged, TNM group, gender, PS and arm of treatment. The proportional hazards assumption was tested for all models. This assumption was not met by the gender univariate Cox model and a Royston–Parmar flexible parametric model was fitted for this case. As with the Cox model, the Royston–Parmar gender model was non-statistically significant with an almost identical hazard ratio (HR). Therefore, for simplicity only the results of the gender Cox model have been reported. Significant parameters in univariate analysis were included in a multivariate Cox analysis. Multivariate models were compared using the Akaike Information Criteria (AIC) and the Bayesian Information Criteria (BIC). Both criteria select the best-fit model as the one minimising the AIC and BIC scores. All statistical analyses were carried out in R version 3.2.3 with *P* values of ≤0.05 considered significant. This analysis was exploratory. Based on the difference in survival observed in this sample (*n* = 75), 15 patients in the unfavourable group provide 80% power with a one sided significance level of 0.05.

## Results

### Patient demographics

A total of 75 patients were included in this analysis. This was due to a lack of funding to expand to all sites. Of note grant funding was sought at the time of the inception of this study in 2008 but declined due to a lack of published data on CTCs in SCLC. Due to the data that were emerging from our own centre and elsewhere we were able to secure local funds to generate exploratory data in Manchester. Baseline characteristics of CTC-tested patients and the overall CONVERT population are summarised in Table 1. The overall population comprised of a greater proportion of PET staged patients (CTC subpopulation 36% versus overall population 57%). As PET staging was not mandated in CONVERT, these differences were assumed within the expected variations observed between participating centres.

**Table 1.** Patient characteristics of CTC subpopulation and overall CONVERT population

	CTC subpopulation				Overall population	
	*N*	%	Arm 1	Arm 2	Arm 1	Arm 2
No. patients	75	100	39	36	273	274
Age at baseline, years, median (range)	62.7		65.8	61.6	63.8	62.5
	(77.1–45.1)		(51.5–77.1)	(45.1–76.8)	(34.2–81.6)	(29.7–84.3)
Sex						
Female	40	53	20 (51%)	20 (56%)	123 (45%)	127 (46%)
Male	35	47	19 (49%)	16 (44%)	150 (55%)	147 (54%)
TNM group						
Group 1: I–II	11	15	7 (18%)	4 (11%)	51 (19%)	35 (13%)
Group 2: III	61	81	30 (77%)	31 (86%)	207 (76%)	219 (80%)
ECOG PS						
0	22	29	12 (31%)	10 (28%)	123 (45%)	125 (46%)
1	49	65	25 (64%)	24 (67%)	142 (52%)	137 (50%)
2	4	5	2 (5%)	2 (6%)	8 (3%)	9 (3%)
PET						
Staged	27	36	14 (39%)	13 (33%)	157 (57%)	155 (57%)
Non-staged	48	64	22 (61%)	26 (67%)	113 (41%)	118 (43%)
CTC count, median (range)			1 (0–3750)			
Per arm, median (range)			1 (0–164)	1 (0–3750)		

CTC, circulating tumour cell; TNM, tumour node metastasis; ECOG PS, Eastern Cooperative Oncology Group Performance Status; PET, positron emission tomography.

### Association of CTC count with clinical characteristics

CTCs were detected in 60% (45/75) of patients with a median of 1 (range 0–3750). There was a non-significant trend to higher CTC count in TNM stage III versus TNM stage I–II patients, (*P* = 0.081; supplementary Figure S1A, available at *Annals of Oncology* online). Similarly, a non-significant trend to higher CTC count in PS 1 versus PS 0 patients (*P* = 0.088; supplementary Figure S1B, available at *Annals of Oncology* online) was observed. A statistically significant but weak correlation was found between CTC count and GTV (*r* = 0.3495, *P* = 0.00716; supplementary Figure S2, available at *Annals of Oncology* online).

Clinical characteristics and association with CTC count thresholds of <2 versus ≥2, <15 versus ≥15 and <50 versus ≥50 are shown in Table 2. Female gender was significantly associated with a CTC count of ≥2 (*P* = 0.0345) and ≥15 (*P* = 0.0017). A CTC count of ≥15 was significantly associated with PS 1 versus PS 0 (*P* = 0.0019). Age, treatment arm, TNM stage and PET staging were not significantly associated with any of the CTC thresholds.

**Table 2.** Prevalence of CTC and association of CTC thresholds with clinical characteristics (*N* = 75)

	2-CTCs threshold		15-CTCs threshold		50-CTCs threshold	
Variable	<2 CTCs (*N*=43)	≥2 CTCs (*N*=32)	<15 CTCs (*N*=58)	≥15 CTCs (*N*=17)	<50 CTCs (*N*=67)	≥50 CTCs (*N*=8)
Age at baseline, years, median (range)	61.2	63.7	61.2	64.3	62.7	63.5
	(48.8–77.1)	(45.1–76.4)	(45.1–77.1)	(59.1–71.0)	(45.1–77.1)	(59.1–71.0)
Mann–Whitney's *P*	0.8639		0.1818		0.6492	
Sex						
Female	18	22	25	15	33	7
Male	25	10	33	2	34	1
Fisher's exact *P*	0.0354		0.0017		0.0606	
TNM group						
Group 1: I–II	9	2	10	1	10	1
Group 2: III	32	29	46	15	55	7
Fisher's exact *P*	0.1004		0.4360		1	
ECOG PS						
0	15	7	22	0	22	0
1	27	22	34	15	42	7
2	1	3	2	2	3	1
Fisher's exact *P*	0.2906		0.0019		0.0884	
PET						
Staged	7	10	14	3	16	1
Non-staged	35	17	42	10	47	5
Fisher's exact *P*	0.0850		1		1	
Arm						
Arm 1	24	15	32	7	36	3
Arm 2	19	17	26	10	31	5
Fisher's exact *P*	0.4897		0.4099		0.4692	

CTCs, circulating tumour cells; TNM, tumour node metastasis; ECOG PS, Eastern Cooperative Oncology Group Performance Status; PET, positron emission tomography.

### Prognostic significance of CTC count

**Figure 1.**
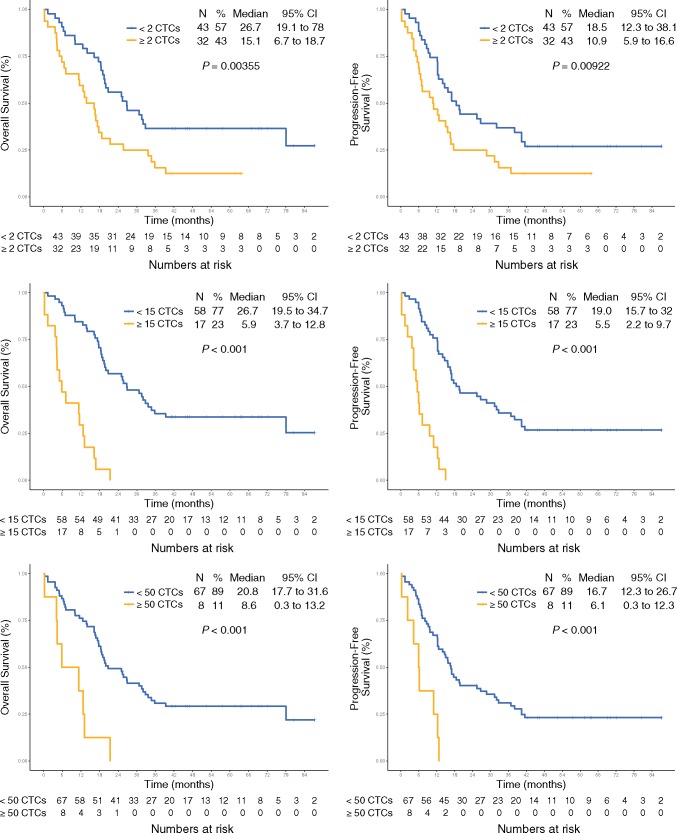
Kaplan–Meier curves for overall survival (OS) and progression-free survival (PFS) for studied thresholds of circulating tumour cells (CTC)/7.5 ml of blood: <2 and ≥2 CTCs (A, B); <15 and ≥15 CTCs (C, D) and <50 and ≥50 CTCs (E, F).

The optimal CTC count threshold for LS-SCLC was defined as the count with the most significant log-rank test split with Bonferroni correction. This method identified that a threshold of 15 CTCs separated patients into optimal ‘favourable (<15 CTCs)’ and ‘unfavourable’ (≥15 CTCs)’ prognostic groups. The median PFS for patients with <15 CTCs was 19.0 months (95% CI 15.7–32.0, *n* = 58) whereas for patients with ≥15 CTCs the median PFS was 5.5 months (95% CI 2.2–9.7, *n* = 17). The median OS for <15 CTCs was 26.7 m (95% CI 19.5–34.7) compared with 5.9 m (95% CI 3.7–12.8) for versus ≥15 CTCs (Figure 1C and D).

Previously published CTC count thresholds of 2 and 50 CTCs were validated in this cohort and correlated significantly with PFS and OS (Figure 1A, B, E and F). For the CTC count threshold of <2 versus ≥2 CTCs, the median PFS was 18.5 months (95% CI 12.3–38.1) versus 10.9 months (95% CI 5.9–16.6) and the median OS 26.7 months (95% CI of 19.1–78) versus 15.1 months (95% CI 6.7–18.7), respectively. In patients with <50 versus ≥50 CTCs the median PFS was 16.7 months (95% CI 12.3–26.7) versus 6.1 months (95% CI 0.3–12.3) and the median OS was 20.8 months (95% CI 17.7–31.6) versus 8.6 months (95% CI 0.3–13.2), respectively.

### Univariate and multivariate Cox proportional hazards regression analysis

CTC thresholds of 2, 15 and 50 CTCs were also significant in Cox univariate analysis for PFS (2 CTCs: HR 1.83, 95% CI 1.10–3.07, *P* = 0.021; 15 CTCs: HR 7.09, 95% CI 3.64–13.83, *P*≤0.001; 50 CTCs: HR 4.36, 95% CI 1.96–9.68, *P*≤0.001) and OS (2 CTCs: HR 2.10, 95% CI 1.23–3.58, 0.006; 15 CTCs: HR 7.35, 95% CI 3.77–14.33, *P*≤0.001; 50 CTCs: HR 4.28, 95% CI 1.94- 9.44, *P*≤0.001). PS 1 was the only significant clinical factor significant for both PFS (HR 2.16, 95% CI 1.17–3.97, *P* = 0.014) and OS (HR 2.29, 95% CI 1.19 to −4.39, *P* = 0.013) compared with PS 0 as the reference (supplementary Table S1, available at *Annals of Oncology* online). There was no significant difference between PS 2 versus PS 0 groups, due to small PS 2 numbers (*n* = 2).

Multivariate analysis (supplementary Table S2, available at *Annals of Oncology* online) was conducted for 2, 15 and 50 CTC thresholds, adjusting for PS as the only significant clinical factor found in univariate analysis. The 15 CTC threshold emerged as an independent prognostic factor given PS remained a significant prognostic factor for 2 and 50 CTC thresholds but had no additional impact on the 15 CTC threshold model.

The 15 CTC count threshold model obtained the minimal values for AIC and BIC for OS (AIC 393.95, BIC 395.98) and PFS (AIC 415.03, BIC 417.10) in comparison to the model adjusted for PS (OS: AIC 395.56, BIC 401.64; PFS: AIC 416.91, BIC 423.14) and the 2 and 50 CTC threshold models. Applying the optimal model, the presence of ≥15 CTCs at baseline predicted ≤2 year survival in 100% and ≤1 year survival in 70% of patients.

## Discussion

We previously identified pre-treatment CTC count to be an independent prognostic factor for survival in a mixed population of LS- and ES-SCLC. Here, we have explored their clinical significance in an exclusively LS-SCLC population enrolled within CONVERT, an international phase III clinical trial of curative-intent CCRT. To our knowledge, this analysis is the largest dataset within a randomised controlled trial to demonstrate the prognostic significance of baseline CTC count specific to LS-SCLC patients.

Consistent with prior reports [10, 12], baseline CTC counts of ≥2, ≥15 and ≥50 CTCs are significant for worse PFS and OS. An optimal CTC count threshold of 15 CTCs defined LS-SCLC patients into two distinct prognostic risk groups. For patients with ≥15 CTCs (17/75, 23%), survival was limited to ≤1 year in 70% and ≤2 years in 100% of patients with a median PFS and OS of 5.5 and 5.9 months, respectively. This analysis establishes ≥15 CTC count as an independent prognostic marker in LS-SCLC, irrespective of other clinical variables.

Due to the low prevalence of detectable peripheral blood CTCs in most early stage cancers [18–21], prognostic CTC enumeration has been difficult to implement and validate outside of metastatic disease. In contrast, 60% (*n* = 45/75) of patients had detectable CTCs are baseline in this exclusively LS-SCLC population. Whilst this may reflect the high proportion of stage III (81%) versus stage I–II (15%) patients with detectable CTCs, importantly the prevalence of ≥2, ≥15 and ≥50 CTCs were not significantly associated with TNM stage, PET staging, patient age or treatment arm. The impact of PET staging was analysed in the entire CONVERT population, with no significant PFS or OS difference detected between patients staged by PET versus conventional imaging alone (PFS HR 0.87; 95% CI 0.71–1.07, *P* = 0.198; OS HR 0.87; 95% CI 0.7–1.08; *P* = 0.192) [22]. The high CTC prevalence in LS-SCLC affords future opportunities for CTC-derived explants, CTC culture and ‘switch’ clinical trials in which treatment change is initiated on the basis of longitudinal CTC values. Importantly, although clinical assessment of CTC number is not routine it would be feasible to do this. Running the sample on the analyser, interpreting the result and issuing a report requires a minimum of three working days and is therefore achievable with a turnaround time of <3 weeks in a clinical workflow.

Based upon these findings, we hypothesise that the presence of detectable CTCs in LS-SCLC represents more aggressive intrinsic disease biology with metastatic propensity and advanced disease, even in the absence of extra-thoracic measurable disease. Efforts to define the spectrum of molecular aberrations of CTCs are in progress to better comprehend the metastatic potential of these cells and their intrinsic resistance to chemotherapy and radiotherapy [24, 25].

A major barrier to the routine deployment of CTC count in treatment decision making is a lack of consensus on the optimal CTC count to apply in the clinic. In the present study, the thresholds of 2 and 50 CTCs were based upon previous published series that demonstrated worse median OS in patients with ≥2 CTC compared with <2 CTCs (3.9 versus 14.8 months, *P* < 0.007) [10] and ≥50 compared with <50 CTCs (5.4 versus 11.5 months; P < 0.001) [12]. Whilst the same CTC count thresholds maintained prognostic significance when applied to this exclusive LS-SCLC population, a considerable improvement in median OS was observed within this cohort (≥2 versus <2 CTCs 15.1 versus 26.7 m; ≥50 versus <50 CTCs 8.6 versus 20.8 m) purely on the basis of excluding patients with extensive disease. These results highlight the importance of establishing prognostic CTC count thresholds that are disease and stage specific. Here, the optimal threshold of 15 CTCs improved further on the separation of the favourable and unfavourable prognostic groups.

In this exploratory analysis, a number of limitations should be acknowledged. First, enrolment to the CTC subpopulation was restricted to one major study site with access to and expertise in CTC analysis. Secondly, as serial sampling was not undertaken in this analysis, the pharmacodynamic role of CTCs in LS-SCLC was not investigated. In previous studies, both CTC count after one cycle of therapy and persistence of ≥50 CTCs after one cycle of chemotherapy are highly prognostic [12].

CTC count has been employed as an exploratory biomarker to predict and monitor treatment response in several ES-SCLC trials [26–27]. With a number of promising therapeutics currently under evaluation in SCLC including immunotherapy combinations, rovalpituzumab tesirine (Rova-T), lurbinectedin and poly-ADP ribose polymerase inhibitors, there is a clear unmet need to develop improved maintenance strategies and trial novel combinations in LS-SCLC. From this analysis, prognostic CTC count may also identify patients with the highest probability of achieving a disease-free interval and cure with CCRT alone, rationalising treatment particularly in patients with borderline fitness or at increased risk of toxicity. The presence of CTCs on completion of CCRT could also be of relevance for identifying patients who stand to benefit from maintenance therapy.

In summary, we substantiate previous reports of CTC count as an independent prognostic factor in LS-SCLC. In this analysis, the presence of ≥15 CTCs in a good PS, LS-SCLC patient identifies a high-risk group that has poor survival outcomes despite curative-intent treatment. The detection of ≥15 CTCs in LS-SCLC provides useful prognostic insight to improve disease stratification beyond clinical factors alone with potential future therapeutic application in patient selection for maintenance therapy and rationalising treatment in patients with the highest probability of achieving a disease-free interval and cure with CCRT alone.

## Supplementary Material

mdz122_Supplementary_Data
