# Supplementary figures and images for: Prognostic value of circulating tumour cells in limited-stage small-cell lung cancer: analysis of the concurrent once-daily versus twice-daily radiotherapy (CONVERT) randomised controlled trial
Source: Ann Oncol. 2019 Apr 24;30(7):1114–20. doi: 10.1093/annonc/mdz122 (PMC6637373; doi:10.1093/annonc/mdz122)

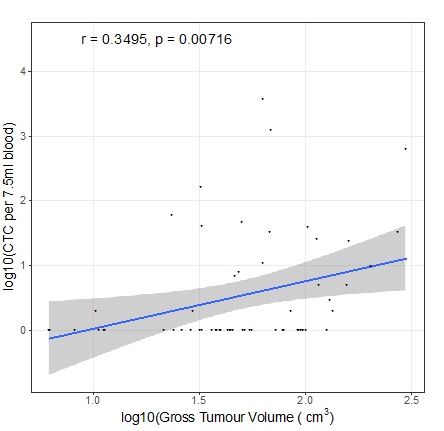

Supplement: mdz122_Supplementary_Data [file mdz122_supplementary_data.zip › mdz122-Suppl_data/Supplementary Figure S2.jpg]

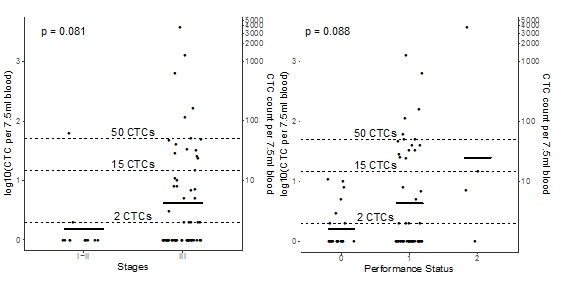

Supplement: mdz122_Supplementary_Data [file mdz122_supplementary_data.zip › mdz122-Suppl_data/Supplementary Figure S1.jpg]
