# Supplementary material for: Prognostic value of circulating tumour cells in limited-stage small-cell lung cancer: analysis of the concurrent once-daily versus twice-daily radiotherapy (CONVERT) randomised controlled trial
Source: Ann Oncol. 2019 Apr 24;30(7):1114–20. doi: 10.1093/annonc/mdz122 (PMC6637373; doi:10.1093/annonc/mdz122)
Supplement: mdz122_Supplementary_Data [file mdz122_supplementary_data.zip › mdz122-Suppl_data/Supplementary Table S1.doc]

Supplementary Table S1: Univariate Analysis

|  | **At-Risk Group** | | **PFS** | | | **OS** | | |
| --- | --- | --- | --- | --- | --- | --- | --- | --- |
| Variable | Positive | Negative | P | HR | 95% CI | P | HR | 95% CI |
| Gender | Female | Male | 0.293 | 1.32 | 0.79 to 2.20 | 0.263 | 1.35 | 0.80 to 2.29 |
| ECOG PS | 1 | 0 | 0.014 | 2.16 | 1.17 to 3.97 | 0.013 | 2.29 | 1.19 to 4.39 |
|  | 2 | 0 | 0.105 | 2.52 | 0.82 to 7.73 | 0.065 | 2.91 | 0.94 to 9.05 |
| TNM | Group 2: III | Group 1: I-II | 0.351 | 1.43 | 0.68 to 3.02 | 0.357 | 1.45 | 0.66 to 3.22 |
| PET Staging | YES | NO | 0.985 | 1.01 | 0.59 to 1.71 | 0.828 | 0.94 | 0.54 to 1.63 |
| Arm | 2 | 1 | 0.403 | 1.25 | 0.74 to 2.08 | 0.453 | 1.22 | 0.72 to 2.07 |
| 2-CTC | ≥ 2 | < 2 | 0.021 | 1.83 | 1.10 to 3.07 | 0.006 | 2.10 | 1.23 to 3.58 |
| 15-CTC | ≥15 | < 15 | < .001 | 7.09 | 3.64 to 13.83 | < .001 | 7.35 | 3.77 to 14.33 |
| 50-CTC | ≥50 | < 50 | < .001 | 4.36 | 1.96 to 9.68 | < .001 | 4.28 | 1.94 to 9.44 |

CTC, circulating tumour cell; TNM, Tumour Node Metastasis; ECOG PS, Eastern Cooperative Oncology Group Performance Status; PET, Positron Emission Tomography
