# Supplementary material for: Prognostic value of circulating tumour cells in limited-stage small-cell lung cancer: analysis of the concurrent once-daily versus twice-daily radiotherapy (CONVERT) randomised controlled trial
Source: Ann Oncol. 2019 Apr 24;30(7):1114–20. doi: 10.1093/annonc/mdz122 (PMC6637373; doi:10.1093/annonc/mdz122)
Supplement: mdz122_Supplementary_Data [file mdz122_supplementary_data.zip › mdz122-Suppl_data/Supplementary Table S2.doc]

Supplementary Table S2: Multivariate Analysis

|  | **At-Risk Group** | | **PFS Risk** | | | | | **OS Risk** | | | | |
| --- | --- | --- | --- | --- | --- | --- | --- | --- | --- | --- | --- | --- |
| Models | Positive | Negative | P | HR | 95% CI | AIC | BIC | P | HR | 95% CI | AIC | BIC |
| With 2-CTCs threshold | | | | | | 434.75 | 440.98 |  | | | 411.57 | 417.65 |
| 2 CTCs at baseline | ≥ 2 | < 2 | 0.021 | 1.85 | 1.10 to 3.12 |  | | 0.006 | 2.15 | 1.25 to 3.70 |  | |
| ECOG PS | 1 | 0 | 0.011 | 2.20 | 1.19 to 4.06 |  | | 0.009 | 2.39 | 1.24 to 4.60 |  | |
|  | 2 | 0 | 0.184 | 2.15 | 0.69 to 6.63 |  | | 0.148 | 2.32 | 0.74 to 7.29 |  | |
| **With 15-CTCs threshold** | | | | | | **416.91** | **423.14** |  | | | **395.56** | **401.64** |
| 15 CTCs at baseline | ≥15 | < 15 | <.001 | 6.03 | 3.00 to 12.08 |  | | <.001 | 6.19 | 3.08 to 12.42 |  | |
| ECOG PS | 1 | 0 | 0.157 | 1.60 | 0.84 to 3.05 |  | | 0.168 | 1.626 | 0.81 to 3.25 |  | |
|  | 2 | 0 | 0.439 | 1.58 | 0.50 to 5.02 |  | | 0.223 | 2.05 | 0.64 to 6.56 |  | |
| With 50-CTCs threshold | | | | | | 432.51 | 438.74 |  | | | 411.97 | 418.04 |
| 50 CTCs at baseline | ≥ 50 | <50 | 0.002 | 3.589 | 1.57-8.18 |  | | 0.003 | 3.42 | 1.52to 7.67 |  | |
| ECOG PS | 1 | 0 | 0.034 | 1.96 | 1.05 to 3.65 |  | | 0.034 | 2.05 | 1.05to 3.97 |  | |
|  | 2 | 0 | 0.298 | 1.85 | 0.58 to 5.89 |  | | 0.148 | 2.35 | 0.74to 7.47 |  | |

CTC, circulating tumour cell; PFS, progression free survival, OS, overall survival, ECOG PS, Eastern Cooperative Oncology Group Performance Status
